# Proximal femoral excision with interposition myoplasty for cerebral palsy patients with painful chronic hip dislocation

**DOI:** 10.1007/s11832-015-0662-z

**Published:** 2015-06-28

**Authors:** Nirav K. Patel, Sanjeeve Sabharwal, Christopher R. Gooding, Aresh Hashemi-Nejad, Deborah M. Eastwood

**Affiliations:** The Catterall Unit, The Royal National Orthopaedic Hospital, Stanmore, Middlesex, HA7 4LP UK; Department of Surgery and Cancer, Imperial College London, St Mary’s Hospital, London, W2 1NY UK; Department of Trauma and Orthopaedic Surgery, Addenbrooke’s Hospital, Cambridge, CB2 0QQ UK

**Keywords:** Proximal femoral excision, Salvage, Cerebral palsy, Chronic hip dislocation, Interposition myoplasty

## Abstract

**Purpose:**

Proximal femoral excision is a salvage procedure for painful chronic hip dislocation in cerebral palsy (CP) patients. The primary objective of this article is to describe our experience of an amplified interposition myoplasty, with appropriate peri-operative pain and tone management strategies, in a cohort of non-ambulatory CP patients with painful chronic hip dislocation. Our secondary objective is to present the clinical outcomes of these patients.

**Methods:**

We describe our experience in 20 CP patients (25 procedures) at mean 54-month (range 27–169) follow-up with a surgical technique that includes an augmented interposition myoplasty and tone management. The indications for surgery were pain (21 hips), poor sitting tolerance (11) and difficulty with perineal care (8).

**Results:**

The mean age was 22 years (range 10–40) with 11 patients Gross Motor Function Classification Scale (GMFCS) IV and 9 patients GMFCS V. Mean length of stay was 13 days (3–35). One procedure required revision at 12 months. Mean pain score improved from 7.8 (5–10) pre-operatively to 2.8 (1–5) post-operatively (*p* < 0.001). Sitting tolerance improved in all patients and in 75 % (15) perineal care was easier.

**Conclusions:**

Our interposition myoplasty technique with individualised pain/tone management has good outcomes in this cohort of patients with multiple co-morbidities.

**Electronic supplementary material:**

The online version of this article (doi:10.1007/s11832-015-0662-z) contains supplementary material, which is available to authorized users.

## Introduction

Despite preventative screening programmes in some countries [1], in many others [2–5], chronic hip dislocation occurs in up to 75 % of the most severely affected non-ambulant cases of cerebral palsy (GMFCS IV/V) [6–8]. This is related to muscle imbalance and spasticity, as well as immobility and excessive femoral anteversion [9]. Early soft tissue release and femoral and/or pelvic osteotomies may prevent progressive subluxation during childhood that, when untreated, often culminates in frank dislocation with or without symptoms [1, 2, 5, 8].

There are many problems associated with chronic hip dislocation, most importantly pain in 25–70 % of patients [5, 7, 8, 10, 11]. In addition, patients have difficulty with perineal care due to muscle spasticity and contracture; and sitting balance due to joint contracture, pelvic obliquity and scoliosis [2, 8, 12]. All these issues can contribute to sleep disturbance and adversely affect the quality of life of both the patient and their caregivers.

There are several options to treat this condition including femoral resection with subtrochanteric valgus osteotomy [13], open reduction and joint reconstruction [14, 15], arthrodesis [16] and proximal femoral resection [6, 12, 17, 18]. Proximal femoral resection below the lesser trochanter is a particularly well-known salvage procedure, and the technique has evolved to include interposition myoplasty [12, 18]. A proximal femoral resection results in a mobile lower limb that may then be positioned easily and comfortably irrespective of pelvic obliquity or fixed contracture of the contralateral hip. The interposition myoplasty aims to reduce the risk of failure associated with painful proximal femoral migration. Overall, this procedure is associated with an improvement in quality of life and patient/carer satisfaction [12, 18]. However, soft tissue interposition may be suboptimal or often not performed altogether from both our own experience and published reports [6, 17] of continued/recurrent pain.

The majority of patients undergoing this type of surgery have spastic total body involvement cerebral palsy. Pain and muscle spasm are frequently major problems in the early post-operative period, before the true benefits of proximal femoral excision and interposition myoplasty can be realised. As a result, various management strategies should be employed to control these symptoms including the use of analgesic medications, anxiolytics, botulinum toxin injections and skin traction [35]. We know of no study in which a valid and accepted quality of life measure has been used to describe the outcome of salvage surgery for hip dislocation in young patients with CP.

The primary objective of this article is to describe our experience of an amplified interposition myoplasty, with appropriate peri-operative pain and tone management strategies, in a cohort of non-ambulatory CP patients with painful chronic hip dislocation. Our secondary objective is to present the clinical outcomes of these patients.

## Methods

We reviewed a consecutive series of non-ambulatory GMFCS IV/V CP patients who underwent proximal femoral excision with interposition myoplasty between 1998 and 2012 at our tertiary referral centre. The procedures were performed by the three senior authors (DME, AHN and CG). The mean follow-up was 54 months (27–169) and no patients were lost to follow-up. Medical records and radiographs were examined retrospectively for baseline demographics, past medical history (scoliosis, pelvic obliquity, previous hip and/or spine surgery, gastrostomy and use of regular tone reducing medication) (Table 1) and indications for surgery. Where the patient could not communicate, information was gathered from the primary care-giver.

**Table 1:** Associated medical and surgical issues in this patient cohort

	Number of patients
Existing scoliosis and/or pelvic obliquity	14
Previous spine surgery	6
Previous hip surgery	7
Percutaneous endoscopic gastrostomy (PEG)	3
Epilepsy	8
Tone reducing medication (e.g. baclofen/trihexyphenidyl)	12
Pre-operative botulinum toxin use (with benefit)	11

The primary indication for surgery in 21 of 25 hips (18 patients) was the relief of pain associated with a chronic hip dislocation (or a previous femoral head excision) in which reconstructive surgery was deemed impossible. In all of these 21 cases, the pain was persistent and significant, interrupting sleep and uncontrolled with extensive non-operative treatments including regular analgesics, repeated botulinum toxin injections into quadriceps/hamstring/adductor muscles (14 cases) and intra-articular local anesthetic/ steroid injections (two patients) when indicated. No patients were able to transfer comfortably because of pain despite the use of hoists. Moreover, no patients were able to utilise a frame for therapeutic standing and thus all patients were wheelchair-based at the time of surgery (including the 11 GMFCS IV patients). Two patients (four hips) were unable to be positioned in a sitting position and a further 11 patients (14 hips) displayed significant difficulties with sitting position and sitting tolerance (less than 1 h) in a wheelchair despite adaptations to seat position and shape/size. None of the remainder could sit for 3 h before a change in position (i.e., removal from wheelchair) was required. Five patients (eight hips) had significant problems with perineal care/dressing due to limb position, contracture and pain.

## Surgical technique

**Fig. 1:**
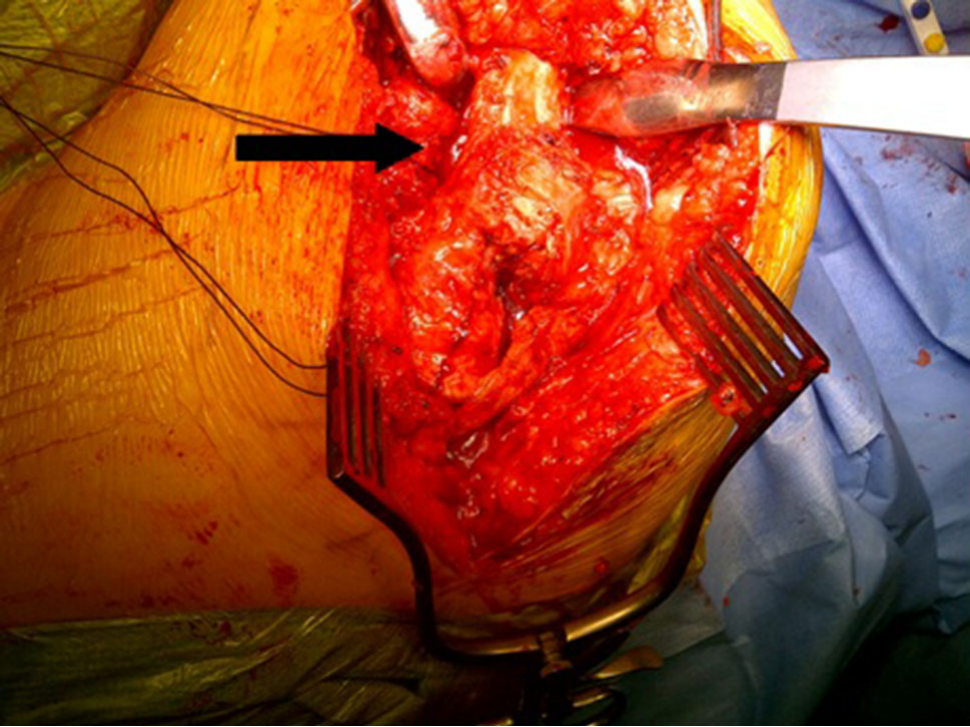
Proximal femur exposed and excised below the level of the lesser trochanter (*black arrow*)

**Fig. 2:**
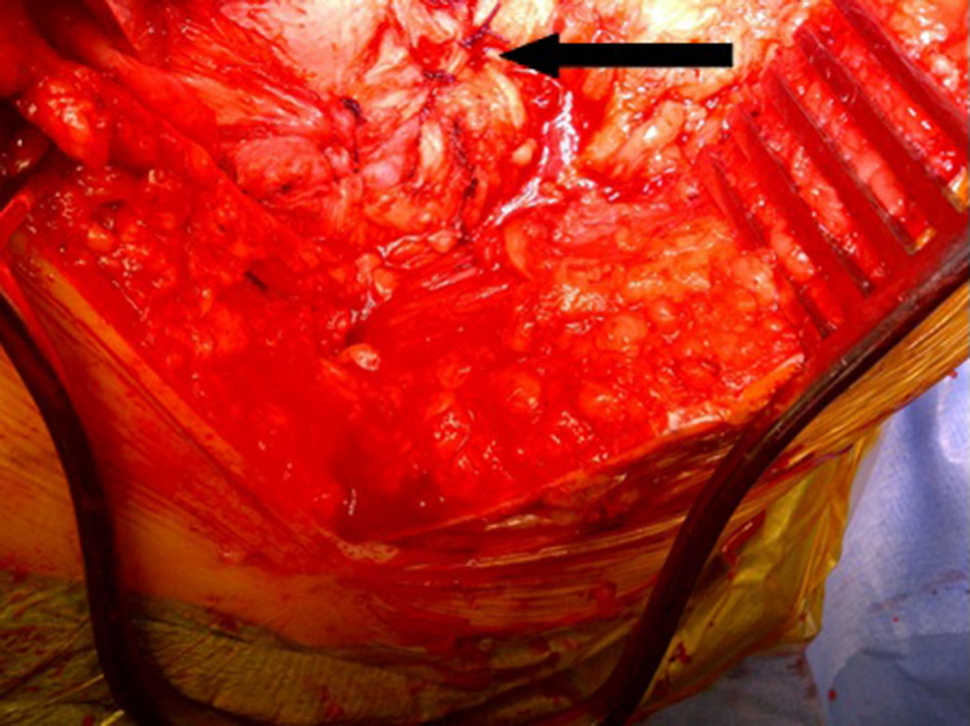
Closure of the capsule over the acetabulum with the psoas sutured to the gluteus medius and minimus (*black arrow*)

**Fig. 3:**
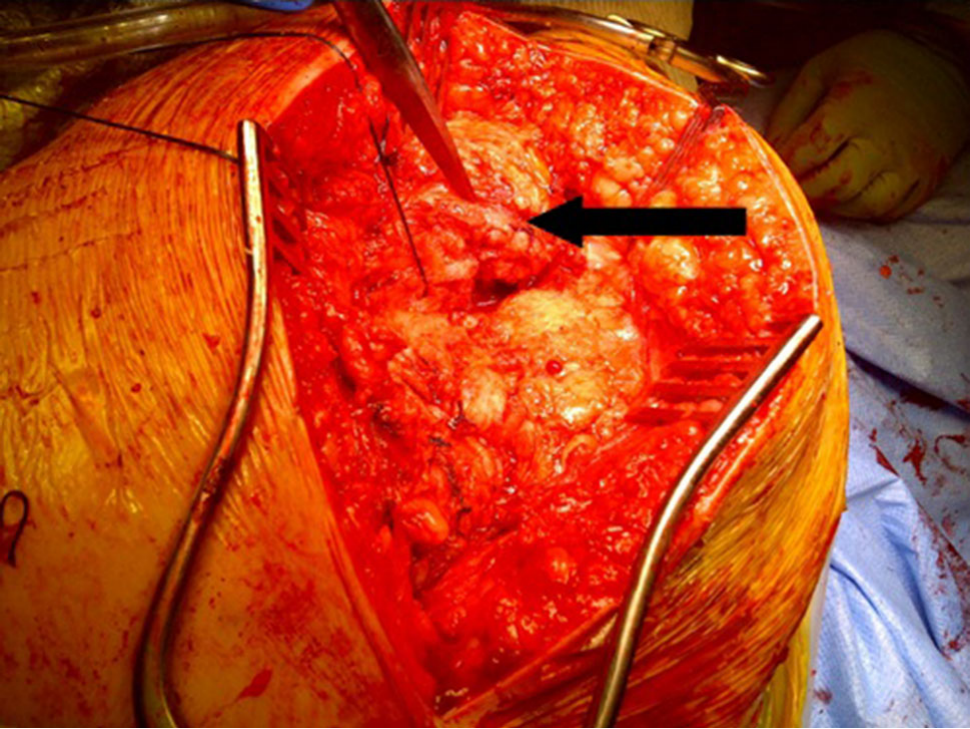
Closure of the vastus medialis and lateralis over the proximal femoral stump (*black arrow*)

**Fig. 4:**
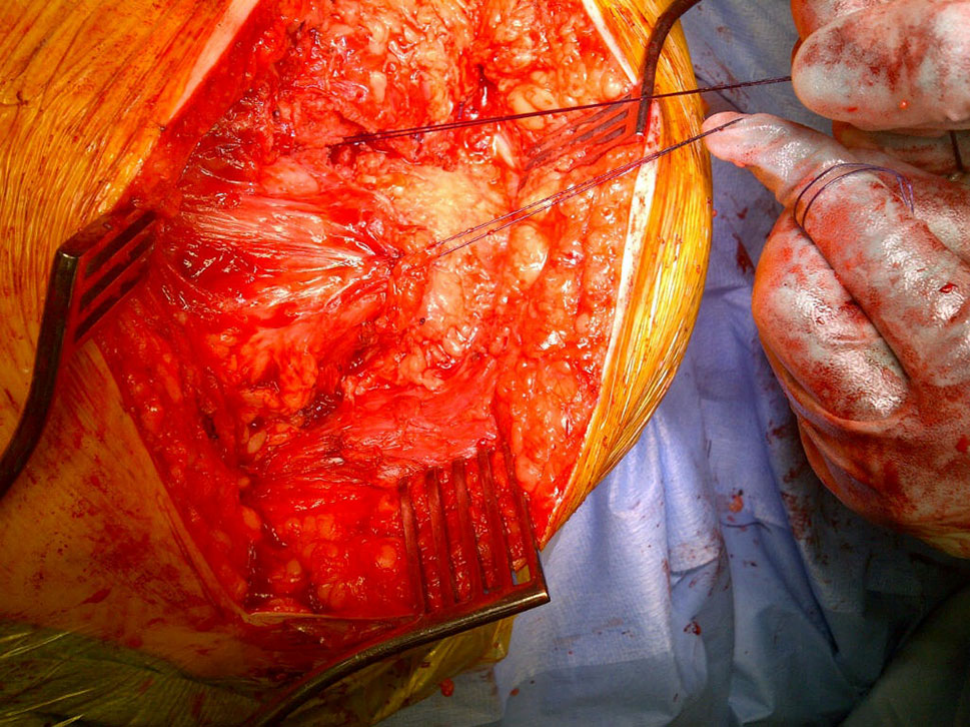
Final result of interposition myoplasty

Under general anaesthesia with local anesthetic and adrenaline skin infiltration, a posterior approach to the hip is followed (see Online Resource). After release of the glutei and vasti from the femur, a capsulotomy and iliopsoas release are performed. The proximal femur is resected 3–4 cm below the lesser trochanter (Fig. 1). Our interposition myoplasty is a modification of the technique described by McCarthy et al. [18]. The acetabulum is covered with a double-breasted capsular repair, and a sling is formed by tenodesing iliopsoas to the glutei, which is sutured to the capsule (Fig. 2), followed by the short external rotators. Drill holes in the proximal femur for transosseus sutures are used to create a muscular envelope using the vasti and medial soft tissues (Fig. 3), resulting in a significant interposition myoplasty (Fig. 4).

All patients received prophylactic intravenous cephalosporin antibiotics on induction, with intra-operative calf pumps, thromboembolic deterrent stockings and low-molecular-weight heparin (in those aged over 16 years) as venous thromboprophylaxis. Post-operative pain and muscle spasm control was tailored to the individual needs, which were often complex. Where possible, patients received an epidural with nurse (or patient if appropriate) controlled intravenous pump analgesia supplemented by routine oral/PEG analgesics. Botulinum toxin injections were used in selected patients with significant pre-operative spasticity. Although not widely described in the literature, we have also found skin traction an effective method of managing selected patients with more significant muscle spasm in the immediate post-operative period. Prophylaxis for heterotopic ossification had not been used routinely in keeping with a recent report [35].

Patients were offered follow-up appointments at 6 weeks, 6 months and annually thereafter. However, as they often lived long distances away from the hospital, we acknowledged that if they and their carers/therapists were pleased with their clinical situation a formal follow-up was not essential. Post-operative complications and the need for revision were recorded. Outcomes for pain [visual analogue scale (VAS) of 0–10, where 0 = no pain and 10 = worst pain ever experienced] were recorded pre- and post-operatively at the latest follow-up clinic visit. Outcomes for function were rated by the patients themselves, including sitting tolerance in a wheelchair (better: more than 3 h, better: up to 3 h, same or worse), problems with perineal care (better, same or worse) and satisfaction (very satisfied, satisfied, dissatisfied or uncertain), and recorded post-operatively at the latest follow-up clinic. If the patients themselves could not communicate, information was gathered from the same primary care-giver where possible. All patients were assessed clinically by the surgeon at 6 weeks, 16 patients were assessed at 6 months and follow-up varied thereafter according to clinical need. Those who could not attend had scores submitted by their local physiotherapist and the most recent follow-up scores were obtained either in clinic or by telephone from an independent junior member of the surgical team.

Radiographic examination included anteroposterior (AP) pelvic radiographs pre-operatively and immediately post-operatively. Further radiographs were only performed according to clinical need (e.g., ongoing pain, functional limitation, problems with perineal care and spinal/pelvic assessment): with assessment of the position of the proximal femur and the presence of heterotopic ossification.

Statistical analysis was performed using SPSS 11.0 (SPSS Inc., Chicago, IL, USA) for Windows. Unless otherwise stated, categorical variables are expressed as percent (frequencies), and continuous variables are expressed as mean (range). For categorical variables, differences between groups were assessed using the Pearson chi-square test or two-tailed Fisher’s exact test. All *p* values were quoted to two decimal places and a *p* value of ≤0.05 was considered statistically significant.

Approval was obtained from the hospital ethics committee.

## Results

Twenty-five procedures were performed in 20 patients (13 female and 7 male) with CP and associated difficulties inherent to that diagnosis (Table 2). The mean age was 22 years (10–40) and all patients were non-ambulatory, GMFCS IV (11 patients) or V (nine patients). The underlying diagnosis in 23 hips was hip dislocation (21 posterior, 2 anterior) with severe erosive changes on the femoral head on radiographs. The female patient with bilateral anterior dislocations was 10 years of age with an inability to flex her hips more than 10°. Two patients had had prior femoral head excisions performed at another centre: in both cases, at operation, the femoral neck was articulating with the pelvis with no soft tissue interposition.

Three patients were given botulinum toxin injections peri-operatively to treat their spasticity: muscles injected included the quadriceps, hamstrings and adductor muscles. All 12 patients on baclofen/trihexyphenidyl had their dose increased for the peri-operative period and diazepam was prescribed as required for post-operative spasm. Of the eight patients who were not taking tone-reducing medication pre-operatively, two who had not taken baclofen previously were prescribed it for the peri-operative period. The remaining six had previously found no benefit with this medication.

Postoperatively, all patients had extremely mobile hip ‘articulations’ due to the femoral shortening inherent with this procedure and the associated soft tissue releases. Nine patients were placed electively in post-operative skin traction because of the severity of their baseline spasticity/dystonia: the traction was used to maintain generalised limb alignment and to reduce spasm. Traction was used for no more than 3–5 days and, during this time, the weights were removed twice daily for physiotherapy range of movement exercises.

All patients received range of movement exercises from the first post-operative day and were placed in their wheelchairs when comfortable at a mean of 3 days post-operatively. Temporary adaptations to their wheelchairs were made at this point and in all patients a formal review of their wheelchair requirements was arranged for 6 weeks post-operatively.

Two patients had significant post-operative pain that delayed their discharge. On discharge they continued with oral muscle relaxants, appropriate analgesic medication and an increased physiotherapy input to maintain limb position.

Two patients had post-operative chest infections requiring admission to the high dependency unit but were treated successfully with antibiotics. No patients required a blood transfusion post-operatively and there were no mortalities.

The mean length of stay was 13 days (3–35 days): all those on skin traction were discharged within 7 days without home traction. The discharge destination was unchanged for all patients: own home [16], residential care [2] or nursing home [2].

One patient had a superficial wound infection that settled with oral antibiotics and one patient was re-admitted to a local hospital for constipation at 3 weeks post-discharge, treated successfully with enemas. The child who had anterior dislocations re-presented at 12 months with one femur protruding through the buttock skin. At revision it was noted that the femoral aspect of the interposition myoplasty had failed (suture failure and button-holing of the proximal femur through soft tissues).

At a mean 54-month (range 27–169) follow-up, information from clinic assessments and physiotherapy reports (supplemented by telephone review in 6 patients) confirmed that ‘hip’ movements had been maintained with flexion from 0–90°, comfortable abduction to at least 20° and free rotation in all patients.

**Fig. 5:**
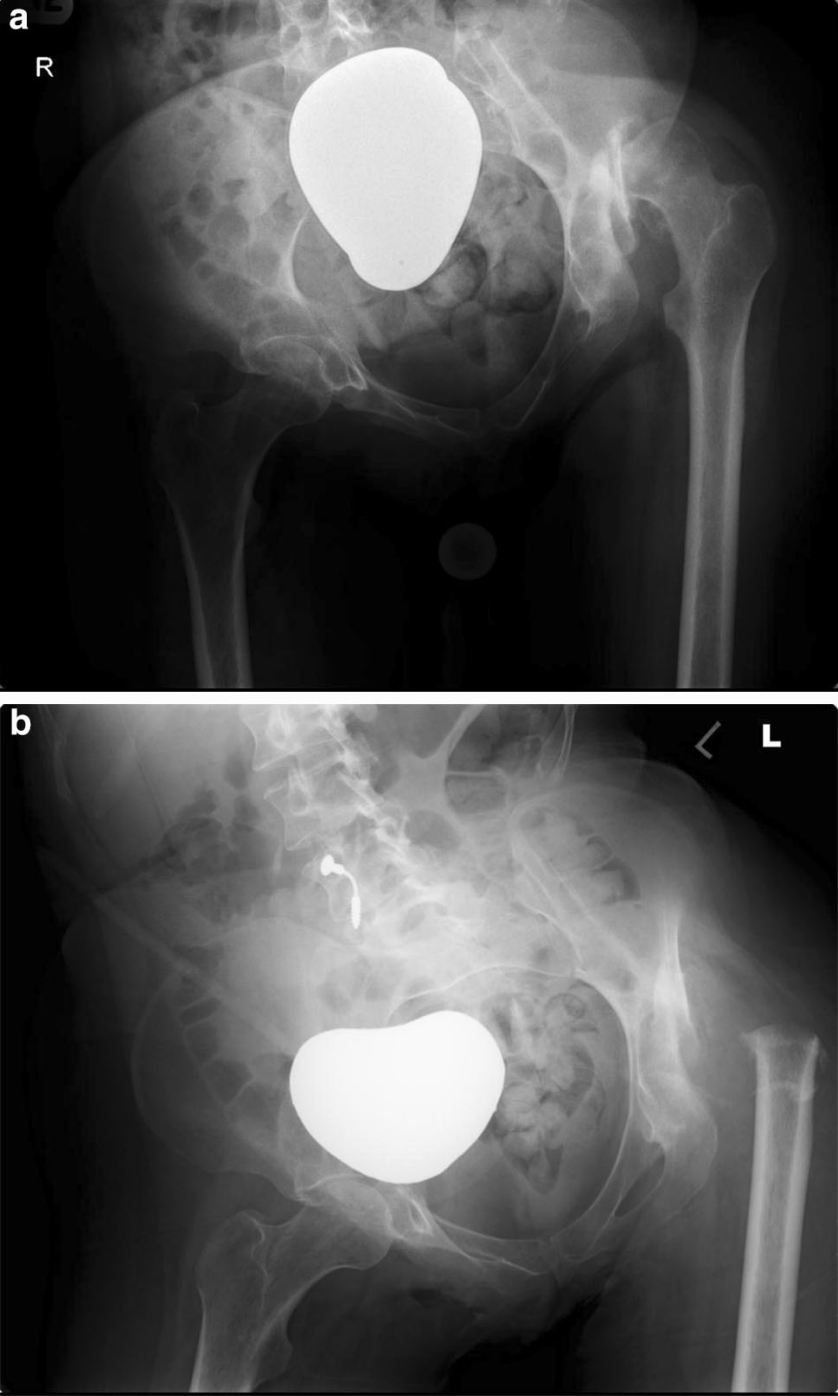
Pre- (**a**) and post-operative (**b**) anteroposterior pelvic radiographs of a 32-year-old female with a painful dislocated left hip and secondary osteoarthritis undergoing a proximal femoral excision

The mean overall VAS pain score had improved from 7.8 (5–10) pre-operatively to 2.8 (1–5) at latest follow-up (*p* < 0.001). There was no significant change at final follow-up in terms of pain score from that seen at 6 months. No patients were taking regular analgesic medication. All patients could sit comfortably in adapted wheelchairs (that accommodated the short limb and maintained a stable pelvis/minimised pelvic obliquity) for more than 1 h and tolerated hoisting with no significant pain. Sixty-five percent of patients could sit comfortably for 3 h or more, which, with a change of position at lunchtime and a further 3 h seated in the afternoon, equates to half a day. Post-operative sitting tolerance and perineal care scores were improved for all patients/carers who reported it as a problem pre-operatively. In addition some patients/carers reported an improvement in these activities even when there had been no perceived problem pre-operatively (Table 3). All patients were either ‘very satisfied’ [11] or ‘satisfied’ [9] with the procedure. Radiological analysis in the immediate post-operative period revealed no significant proximal femoral migration in any patient. Ten patients (12 hips) had undergone further radiographic assessment at follow-up beyond 12 months post-operatively: none of these 12 hips demonstrated proximal femoral migration (Fig. 5), but 3 of 12 hips did show evidence of minor heterotopic ossification.

**Table 2:** Overview of all patients in this study

Patient	Age (years)	Sex	GMFCS	Scoliosis and/or pelvic obliquity (*previous spine surgery)	Use of skin traction	Botulinum toxin (used peri-operatively)	Pre-operative VAS	Post-operative VAS	Follow-up (months)	Complications
1	40	F	5	Y			6	2	36	
2	36	F	4	Y*			5	1	37	
3	16	M	4	Y*			7	4	36	
4	22	F	5	Y			6	2	38	
5	20	F	4	Y	Y		9	5	35	
6	22	M	5	Y		Y	8	4	40	Prolonged pain
7	10	F	4		Y		9	5	68	Chest infection
8	17	F	4		Y	Y	7	1	28	Unilateral revision for proximal femoral migration
9	19	F	4	Y*	Y		8	1	39	
10	38	F	4				9	2	28	Superficial wound infection
11	32	F	4	Y			10	0	49	
12	22	F	5	Y*	Y		7	3	169	
13	23	M	4		Y		7	3	123	Constipation
14	19	M	4	Y	Y	Y	9	3	157	Prolonged pain
15	23	M	4	Y*			10	2	27	
16	26	F	5	Y*			8	1	59	
17	17	M	5	Y	Y		10	1	36	Chest infection
18	15	M	5	Y	Y		10	2	27	
19	19	F	5				8	3	47	
20	31	F	5				9	1	54	

*GMFCS* gross motor function classification scale, *VAS* visual analogue scale of 0–10: where *0* no pain and *10* worst pain ever experienced

**Table 3:** Patient/carer’s subjective impression of the outcome of surgery on sitting tolerance and perineal care

Outcome	Pre-operative rating: perceived problem or not? (number of patients)		Post-operative rating (number of patients)			
	No	Yes	Worse	Same	Better	
Sitting tolerance 1 h or less	7	13	0	0	7 (1–3 h)	13 (>3 h)
Perineal care	15	5	0	5	15

## Discussion

Chronic hip dislocation in non-ambulatory CP patients often causes significant hip pain and functional difficulties [6, 18, 19]. Proximal femoral resection is an established salvage technique for managing these patients because it leaves a pain-free-mobile ‘articulation’. However, symptoms can recur with proximal femoral migration. Like others, we believe that adequate interposition myoplasty is an important part of proximal femoral excision surgery that may reduce failure rates. This, however, must be in addition to appropriate use of peri-operative analgesia, botulinum toxin injections and skin traction. We describe a reproducible surgical technique and management strategy, with a high satisfaction rate, which has improved quality of life in this small series of patients.

Prevention of hip dislocation is important during childhood and a screening programme with early intervention can be effective [1]. This includes soft tissue release such as adductor tenotomies [20, 21] and femoral/pelvic osteotomies in young patients with an open triradiate cartilage and without secondary degenerative change [14, 15, 22, 23]. Patients with a chronic hip dislocation can be free of pain and functional problems but with the onset of degenerative change when there may be erosion of the femoral head, pain tends to become a significant issue provoking spasm and exacerbating existing joint stiffness and contracture. When this occurs with significant symptoms despite non-operative intervention, further surgical intervention is considered. In such cases, the preferred technique would still be surgical reconstruction of the hip if feasible particularly in the younger, skeletally immature patient. There are, however, other options that deserve consideration particularly in the older, non-ambulant patient with CP.

Total hip arthroplasty may have satisfactory outcomes in mobile CP patients [15, 24] but bony deformity, poor bone stock and pelvic obliquity make implant positioning difficult with a high risk of dislocation and revision [11, 25]. It is therefore best avoided in GMFCS level IV and V patients. Valgus subtrochanteric osteotomy [26] may be more successful, although abutment of the proximal femur into the acetabulum and metalwork-associated problems are common [13, 17]. Arthrodesis improves pain and lower limb position with good outcomes [2, 16] but requires unilateral involvement, an ability to ambulate and no spine pathology [27]. Again, this is inappropriate for the GMFCS IV and V patients reported in this paper.

A more radical treatment involves femoral head resection in a ‘Girdlestone-type’ procedure and, although this does relieve pain well [28], there is a risk of painful failure with proximal migration and heterotopic ossification [11]. Thus, a more extensive proximal femoral excision with varying soft tissue interposition techniques has evolved to be a more reliable procedure for these patients [6, 12, 18, 29]. Specifically, it may avoid the poor results reported after a ‘Girdlestone-type’ procedure [30–32]. Traditionally, soft tissue interposition involved simple capsular closure over the acetabulum alone (i.e., no myoplasty) with only short-term outcomes reported [6]. In a study of 14 hips, Castle and Schneider [6] described proximal femoral excision with capsular and fascial acetabular closure. All patients had improved pain, sitting balance and range of movement, attributed to the good soft tissue interposition. Most subsequent studies used a similar technique [17, 18, 29] and achieved equivalent outcomes, although some demonstrated problems with proximal femoral migration despite soft tissue interposition and prolonged skeletal traction [17, 29].

In this series, there was one revision due to migration of the proximal femur but there were no other cases of symptomatic proximal femoral migration and none identified in those undergoing radiological follow-up. We accept there still may be a degree of proximal femoral migration in some of these patients but this is only of concern if it causes pain and/or functional difficulty, including the ability to sit. Our one case of femoral migration was associated with pain due to the protrusion of the bone through the skin and soft tissues where at revision surgery, failure of the myoplasty was noted. This may have been preventable by a more prolonged period of skin traction to control muscle spasm and allow the soft tissues to heal but our philosophy has been to avoid prolonged bed rest with the attendant risks of chest infection and pressure sores.

Adequate soft tissue interposition acts as a mechanical barrier between the proximal femur and the pelvis as well as a cushioning effect to minimise pain, whilst maintaining motion. The two cases of previous femoral head excision performed at another centre were found to have the femoral neck abutting the acetabulum without any soft tissue interposition. We used a modification of the technique by McCarthy et al. [18] who showed good results in all 34 patients, although they recommended the procedure in skeletally mature children to minimise the risk of proximal femoral migration and heterotopic ossification. This has not been replicated in subsequent studies [12], where most patients (70 %), irrespective of skeletal maturity, benefited from surgery, without problems of proximal migration or heterotopic ossification [12]. Our cohort included one skeletally immature child with bilateral anterior dislocations: she was our only case where revision surgery (unilateral) was required.

Clinical outcomes at the latest follow-up improved for all patients in our study. Pain was reduced significantly at follow-up in a similar manner to the results reported by Leet et al. [33], who showed a decrease in VAS score from 8.2 to 2.9. Other studies have shown that pain relief may take up to 6 months to occur post-operatively [12, 29] and two of our patients had prolonged post-operative pain that resolved eventually with analgesics and a continued physiotherapy programme. Sitting balance improved post-operatively in all patients as shown in previous studies [6, 12, 17, 18, 29, 34]. For example, one study showed an improvement in sitting time from 3 to 9 h post-operatively [29]: our cohort improved in a similar fashion with 60 % managing more than 3 h and all managing more than 1 h (in suitably adapted seating). Our sitting outcomes may have been limited because the patients were in severe distress pre-operatively, and other aspects of their CP (e.g., spine, knee and foot positions) also affect their ability to sit for more than 1–3 h comfortably. Similarly, perineal care also improved post-operatively in all patients in accordance with the literature [12, 17, 18, 34]. It is worth noting that carers who rated outcomes as ‘the same’ did not have significant problems with the respective function pre-operatively, although others noted improvement even though they had not recognised a problem pre-operatively.

Heterotopic ossification is a well-recognised complication of proximal femoral excision known to cause pain and require further surgery [12, 17, 18, 34]. Radiographic analysis revealed 3 out of 12 hips (10 patients) with asymptomatic heterotopic ossification in our series. This is unlike studies by Knaus et al. [12] and Abu-Rajab and Bennet [34], where all patients had some degree of heterotopic ossification, albeit not problematic in the majority of cases. This was also found to be the case in a more recent study [35]. A possible explanation for our low incidence of heterotopic ossification is the surgical technique we employed with an extra-periosteal dissection, periosteal excision and meticulous soft tissue care: surgical practice that has been advocated strongly by McCarthy et al. [18].

Post-operative skeletal traction was not used routinely in our study but skin traction was applied to the affected leg for 3–5 days in patients with significant pre-operative spasm. This aims to facilitate movement and ease the transition from bed to wheelchair mobilisation. In contrast, many previous studies routinely employed 3–6 weeks of skeletal traction [17, 18, 29, 30] in order to minimise proximal femoral migration. However, traction has the disadvantage of prolonging hospital stay [12] and may still be associated with proximal migration of up to 2.5 cm [12, 29, 33]. With adequate soft tissue interposition, traction can be used selectively to reduce muscle spasm and to ease limb positioning, thereby promoting early mobilisation. Thus we believe it was helpful in selected patients, but it is not indicated to prevent proximal migration. Muscle relaxants and peri-operative neuromuscular blockade with botulinum toxin also facilitated recovery in selected patients.

The satisfaction rate was high in our study with all patients or caregivers either ‘satisfied’ or ‘very satisfied’ with the surgical outcome. This exceeds the findings of previous studies where the majority were either ‘satisfied’ (73.7 %) [12] or ‘willing to recommend surgery to others’ (73.3 %) [33]. This may be explained by our emphasis not only on good patient selection and counselling regarding outcomes, but also on good soft tissue interposition and avoidance of routine skin traction or immobilisation. As such, we facilitate timely discharge when pain and muscle spasm are adequately controlled.

The limitations of our study include a low patient number (similar to other reported series) that may reduce the strength of the conclusions made. Our patient cohort was heterogeneous in terms of age and contained more GMFCS IV than V patients. We believe this may represent a better ability of GMFCS IV patients to express their pain. Our medium-term follow-up (mean 54 months) may underestimate the number of failures, despite the study being performed on patients over a 14-year period. Our outcome measures were subjective and limited, particularly as many patients lived far away and chose not to attend for routine follow-up but we do not know any valid quality of life measure available for use in this specific patient group that can be applied retrospectively. Finally, we did not measure proximal femoral migration objectively nor did we perform radiographs routinely at the latest follow-up, making inferences on proximal femoral migration and heterotopic ossification difficult. However, the need for revision from painful abutment of the femur onto the acetabulum does serve well as an indicator of success.

## Conclusion

Proximal femoral excision is an effective technique for managing painful chronic hip dislocation in patients with non-ambulatory CP that is refractory to more conservative medical measures. Soft tissue interposition is not always performed according to published techniques, and when it is, it varies from simple capsular closure to complete myoplasty. Interposition myoplasty probably functions as a mechanical barrier that helps to reduce proximal femoral migration and articulation with the iliac wing. We therefore feel that this technique, in addition to appropriate use of peri-operative analgesia, tone-reducing medication and skin traction, helps achieve successful outcomes. We have described our proximal femoral resection surgical technique with our experience of post-operative management that has demonstrated substantial short-term clinical improvement in all patients. Importantly, complications were few and patient satisfaction was high.

## Electronic supplementary material

Supplementary material 1 (DOC 30 kb)
