# Supplementary material for: Proximal femoral excision with interposition myoplasty for cerebral palsy patients with painful chronic hip dislocation
Source: J Child Orthop. 2015 Jun 28;9(4):263–71. doi: 10.1007/s11832-015-0662-z (PMC4549349; doi:10.1007/s11832-015-0662-z)
Supplement: Supplementary file 1 — Supplementary material 1 (DOC 30 kb) [file 11832_2015_662_MOESM1_ESM.doc]

**Proximal femoral excision with interposition myoplasty for cerebral palsy patients with painful chronic hip dislocation**

N K PATEL FRCS^1^, S SABHARWAL MRCS^2^, C R GOODING MD FRCS^3^, A Hashemi-Nejad FRCS^1^, D M Eastwood FRCS^1^

^1^ The Catterall Unit, Royal National Orthopaedic Hospital, Stanmore, London, UK

^2^ Department of Surgery and Cancer, Imperial College, 10th Floor QEQM Building, St Mary's Hospital, London, W2 1NY, UK

^3^ Department of Trauma and Orthopaedic Surgery, Addenbrooke’s Hospital, Cambridge, UK

**Correspondence to:**

Mr Nirav K Patel

Specialist Registrar

The Catterall Unit

Royal National Orthopaedic Hospital

Stanmore

Middlesex, HA7 4LP

UK

Email: niravpatel@doctors.org.uk

**Electronic Supplementary Information**

Surgical Technique

The procedure is performed under general anaesthesia with patient positioned in the lateral decubitus position with the use of side supports. Following skin preparation and sterile draping, the skin is marked longitudinally over the region of the greater trochanter. Local anaesthetic (bupivacaine) and adrenaline (1 in 100,000) is infiltrated into the skin and subcutaneous layers for analgesia and haemostasis (Figure 1).

A posterior approach to the hip is followed with incision of the fascia lata in line with its fibres. Gluteus medius and minimus are detached from their femoral insertions and held with a stay suture. Vastus lateralis and medialis are then reflected from their femoral origins beneath the level of the lesser trochanter. Next the capsule is opened longitudinally and stripped from the inter-trochanteric line (Figure 2), the iliopsoas tendon is identified and held with a stay suture before being released from the lesser trochanter (Figure 3). The proximal femur is then free to be resected extraperiostally 3-4 cm below the level of the lesser trochanter (Figure 4). To minimise the risk of heterotopic bone formation, the periosteum is excised, soft tissues are handled carefully, and residual haematoma and bone debris within muscle are minimized using copious saline lavage.

We used a modification of the soft tissue interposition technique previously described by McCarthy et al (18). They sutured ilipsoas to the lateral capsule and abductors to the medial capsule without creating a sling or tenodesis of short external rotators, and there was no preparation or drilling (transosseus sutures) of the femoral stump. Our technique provides a significant soft tissue barrier to the potential articulation between femur and acetabulum. Firstly, the acetabulum is ‘closed’ using a double-breasted capsular repair. This is then augmented with the construction of a sling, as the iliopsoas tendon is tenodesed to the tendons of the gluteus medius and gluteus minimus (Figure 5), which are sutured to the hip capsule. A further interposition of soft tissue is secured with the tenodesis of the short external rotators to the capsule. To achieve cover of the femoral stump, it is first beveled using a bone file and a large swab is used to protect soft tissues from bone debris (Figure 6). Drill holes (2mm) can be made at the proximal end to facilitate muscle anchorage using transosseous sutures. It is covered by a muscular envelope formed by the joining of vastus lateralis to vastus medialis and medial soft tissue structures (Figure 7). The completed soft tissue interposition using myoplasty is therefore substantial (Figure 8). The wound is then closed in layers from fascia to skin. The technique was modified for the 2 patients who had had a previous proximal femoral excision by avoiding further femoral resection and performing the interposition according to the availability of soft tissue.
